# Coupled Free Vibration of Spinning Functionally Graded Porous Double-Bladed Disk Systems Reinforced with Graphene Nanoplatelets

**DOI:** 10.3390/ma13245610

**Published:** 2020-12-09

**Authors:** Tianyu Zhao, Yu Ma, Hongyuan Zhang, Jie Yang

**Affiliations:** 1 School of Science, Key Laboratory of Ministry of Education on Safe Mining of Deep Metal Mines, Northeastern University, Shenyang 110819, China; zhaotianyu@mail.neu.edu.cn (T.Z.); 2000299@stu.neu.edu.cn (Y.M.); 2 School of Automotive and Transportation, Shenyang Ligong University, Shenyang 110168, China; zhy@sylu.edu.cn; 3 School of Engineering, RMIT University, P.O. Box 71, Bundoora, VIC 3083, Australia

**Keywords:** graphene nanoplatelets, double-bladed disk system, coupled vibration, porosity, spinning

## Abstract

This paper presents, for the first time, the mechanical model and theoretical analysis of free vibration of a spinning functionally graded graphene nanoplatelets reinforced composite (FG-GPLRC) porous double-bladed disk system. The nanocomposite rotor is made of porous metal matrix and graphene nanoplatelet (GPL) reinforcement material with different porosity and nanofillers distributions. The effective material properties of the system are graded in a layer-wise manner along the thickness directions of the blade and disk. Considering the gyroscopic effect, the coupled model of the double-bladed disk system is established based on Euler–Bernoulli beam theory for the blade and Kirchhoff’s plate theory for the disk. The governing equations of motion are derived by employing the Lagrange’s equation and then solved by employing the substructure mode synthesis method and the assumed modes method. A comprehensive parametric analysis is conducted to examine the effects of the distribution pattern, weight fraction, length-to-thickness ratio, and length-to-width ratio of graphene nanoplatelets, porosity distribution pattern, porosity coefficient, spinning speed, blade length, and disk inner radius on the free vibration characteristics of the FG-GPLRC double-bladed disk system.

## 1. Introduction

Spinning bladed disk rotor systems are the core components in many rotary machines in helicopter rotor, ship power propulsion system, engineering agitator, and so on [1,2,3,4,5,6]. By adopting the finite element method, Ma et al. [7] studied the vibration characteristics of bladed disk structure subject to rubbing force at the blade tip. Battiato et al. [8] experimentally investigated the vibration response of the spinning bladed disk system. Bai et al. [9] employed an extremum response surface method-based improved substructural component modal synthesis to study the vibration behavior of a mistuned bladed disk structure. These previous studies showed that a continuous increase in the spinning speed frequently leads to excessive vibration of the system.

It has been well accepted that the use of advanced materials is one of the effective ways to improve the mechanical performance of the bladed disk rotor system to avoid undesired vibration. Owing to its superior mechanical properties, graphene and its derivatives such as graphene nanoplatelets have attracted huge attention from both research and industry communities with numerous efforts on the applications of graphene-based nanomaterials in different fields. Rafiee et al. [10] experimentally found that GPLs have distinct advantages as reinforcing nanofillers over carbon nanotubes at a very low content, which has also been theoretically confirmed [11,12,13,14]. Compared with single-layer graphene, GPLs has comparable elastic modulus, less agglomeration, better dispersion, and much lower cost; hence, they have been widely used in many high-performance composite structures with greatly enhanced structural stiffness. Feng et al. [15] investigated the nonlinear free vibration of GPL reinforced beams. By using the finite element method, Tam et al. [16] and Zhao et al. [17] studied the vibration characteristics of GPL-reinforced beams, with a particular focus on the effects of open edge cracks and trapezoidal plates, respectively. Guo et al. [18] presented a theoretical study on the nonlinear bending of GPL-reinforced plates by employing the element-free IMLS-Ritz method. Wu et al. [19] and Song et al. [20] investigated the vibration behavior of FG annular plates and cracked beams reinforced by GPLs in thermal environments. The in-plane and out-of-plane free vibration performance of an FG-GPLRC arch was discussed by Yang et al. [21].

Due to its light weight and low density, porous metal foam is one of the most promising advanced engineering materials [22,23,24]. Based on the sinusoidal shear deformation theory, Wang et al. [25] investigated free vibrations of FG porous cylindrical shells. Ebrahimi et al. [26] studied the nonlinear vibration of FG porous Timoshenko beams. Wang et al. [27] conducted the vibration analysis of FG porous plates in thermal environments. Both the classical and first-order shear deformation plate theories are used by Kim et al. [28] to study the vibration behavior of FG porous microplates. Recent studies [29,30] also showed that incorporating GPLs into FG porous structures is another avenue for the development of advanced composite structures that are lightweight yet very strong.

It should be noted that all of the existing research works available in the open literature are for a single FG-GPLRC beam, plate, or shell only; no research work has been conducted on the mechanical performance of an FG-GPLRC assembly consisting of two or more structural elements. This paper makes the first attempt to model and analyze the free vibration of a spinning FG porous double-bladed disk system reinforced with GPLs. Based on Euler–Bernoulli beam theory and Kirchhoff’s plate theory, the equations of motion governing the coupled vibration are derived by employing Lagrange’s equation. Then, the substructure mode synthesis method and the assumed modes method are adopted to solve the equations of motion. Special attention is given to the effects of the spinning speed, GPL distribution pattern, GPL weight fraction, length-to-thickness ratio and length-to-width ratio of GPLs, porosity distribution pattern, porosity coefficient, blade length, and disk inner radius on the free vibration of the rotor. The obtained results are of practical significance for the design of FG-GPLRC porous double-bladed disk systems.

## 2. Theoretical Formulations

### 2.1. Modeling

**Figure 1:**
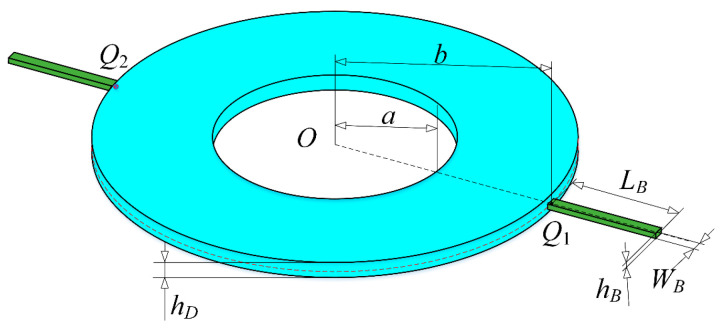
A spinning double-bladed disk system.

The theoretical model of a double-bladed disk system is shown in Figure 1. The two same blades are connected at *Q_k_* (*k* = 1, 2) on the outer edge of the disk in opposite directions with the inner edge of the disk clamped. As can be seen, the inner radius, outer radius, and thickness of the disk are *a*, *b* and *h_D_*, respectively; the length, width, and thickness of the blades are *L_B_*, *W_B_*, and *h_B_*, respectively.

**Figure 2:**
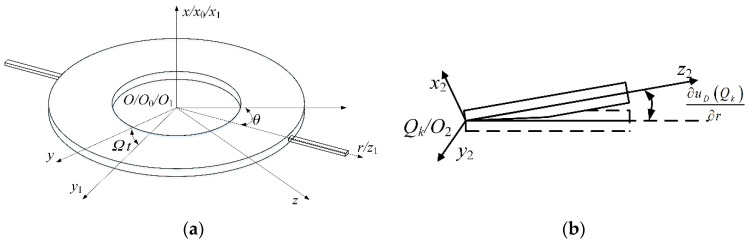
Coordinate systems: (**a**) for the disk, (**b**) for the *k*th blade.

To describe the motion and deformation of the coupled rotor, four different coordinate systems as shown in Figure 2 are defined, where *O*-*xyz* is the global system fixed at *O*; *O*_0_-*x*_0_*rθ* is a cylindrical coordinate system spinning at *Ω* along the *x*_0_-axis; *O*_1_-*x*_1_*y*_1_*z*_1_ is a rectangular coordinate system spinning at *Ω* along the *x*_1_-axis; *O*_2_-*x*_2_*y*_2_*z*_2_ is a rectangular coordinate system fixed at *Q_k_* spanned by a deflection angle along the *y*_1_-axis with respect to *O*_1_-*x*_1_*y*_1_*z*_1_.

### 2.2. Material Properties

**Figure 3:**
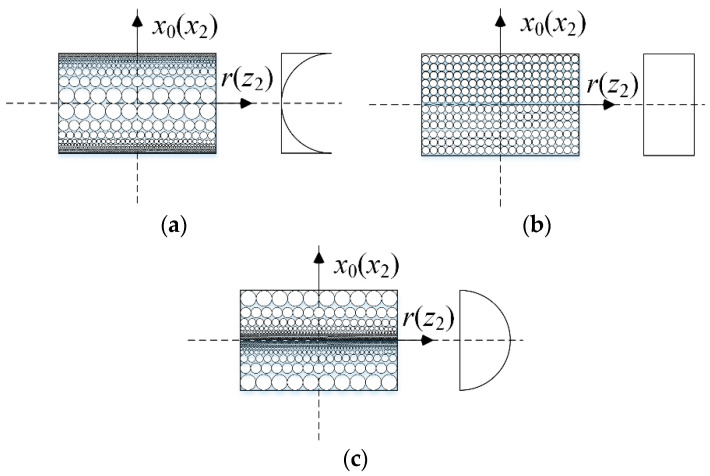
Porosity distribution patterns in the disk and blades: (**a**) Pattern X*_PD_* (X*_PB_*), (**b**) Pattern U*_PD_* (U*_PB_*), (**c**) Pattern O*_PD_* (O*_PB_*).

The double-bladed disk system is made of a porous metal matrix and GPL reinforcement. Three porosity distribution patterns are considered as shown in Figure 3 for the disk (X*_PD_*, U*_PD_*, O*_PD_*) and two blades (X*_PB_*, U*_PB_*, O*_PB_*). According to the open-cell scheme [31], the specific expressions of material properties are determined by:

(1) $$\mathrm{Porosity}\;\mathrm{Pattern}\;X_{PD}\;(X_{PB}):\;\left\{ \begin{array}{l} E_{D(B)}\left(x_{0(2)}\right)=E_{D0(B0)}\left[1-e_{D0(B0)}\cos\left(\frac{\pi x_{0(2)}}{h_{D(B)}}\right)\right]\\ \rho_{D(B)}\left(x_{0(2)}\right)=\rho_{D0(B0)}\left[1-e_{Dm(Bm)}\cos\left(\frac{\pi x_{0(2)}}{h_{D(B)}}\right)\right]\\ \mu_{D(B)}\left(x_{0(2)}\right)=\mu_{D0(B0)} \end{array}\right.$$

(2) $$\mathrm{Porosity}\;\mathrm{Pattern}\;U_{PD}\;(U_{PB}):\;\left\{ \begin{array}{l} E_{D(B)}\left(x_{0(2)}\right)=E_{D0(B0)}\alpha_{D(B)}\\ \rho_{D(B)}\left(x_{0(2)}\right)=\rho_{D0(B0)}\alpha_{D(B)}^{\prime }\\ \mu_{D(B)}\left(x_{0(2)}\right)=\mu_{D0(B0)} \end{array}\right.$$

(3) $$\mathrm{Porosity}\;\mathrm{Pattern}\;O_{PD}\;(O_{PB}):\;\left\{ \begin{array}{l} E_{D(B)}\left(x_{0(2)}\right)=E_{D0(B0)}\left\{1-e^{*}{}_{D0(B0)}\left[1-\cos\left(\frac{\pi x_{0(2)}}{h_{D(B)}}\right)\right]\right\}\\ \rho_{D(B)}\left(x_{0(2)}\right)=\rho_{D0(B0)}\left\{1-e^{*}{}_{Dm(Bm)}\left[1-\cos\left(\frac{\pi x_{0(2)}}{h_{D(B)}}\right)\right]\right\}\\ \mu_{D(B)}\left(x_{0(2)}\right)=\mu_{D0(B0)} \end{array}\right.$$

where subscripts *D* and *B* stand for the disk and blades, respectively; *E_D_*_(*B*)_, *ρ_D_*_(*B*)_, and *μ_D_*_(*B*)_ are the effective Young’s modulus, mass density, and Poisson’s ratio, respectively; *E_D_*_0(*B*0)_, *ρ_D_*_0(*B*0)_, and *μ_D_*_0(*B*0)_ are the Young’s modulus, mass density, and Poisson’s ratio of the GPL-reinforced material without pores, respectively; *e_D_*_0(*B*0)_, e^*^_D__0(*B*0)_, and *α_D_*_(*B*)_ are the porosity coefficients with respect to the three porosity distributions, respectively; *e_Dm_*_(*Bm*)_, e^*^_Dm__(*Bm*)_, and *α′_D_*_(*B*)_ are the mass density coefficients with respect to the three porosity distributions, respectively.

Applying the typical mechanical property relationship

(4) $$\frac{E_{D(B)}}{E_{D0(B0)}}={\left(\frac{\rho_{D(B)}}{\rho_{D0(B0)}}\right)}^{2}$$

gives the relations of the porosity coefficients and mass density coefficients:

(5) $$\left\{ \begin{array}{l} 1-e_{Dm(Bm)}\cos\left(\frac{\pi x_{0(2)}}{h_{D(B)}}\right)=\sqrt{1-e_{D0(B0)}\cos\left(\frac{\pi x_{0(2)}}{h_{D(B)}}\right)}\\ 1-e^{*}{}_{Dm(Bm)}\left[1-\cos\left(\frac{\pi x_{0(2)}}{h_{D(B)}}\right)\right]=\sqrt{1-e^{*}{}_{D0(B0)}\left[1-\cos\left(\frac{\pi x_{0(2)}}{h_{D(B)}}\right)\right]}\\ \alpha_{D(B)}^{\prime }=\sqrt{\alpha_{D(B)}} \end{array}\right..$$

Due to the equal mass in different distributions, it can be obtained as:

(6) $$\left\{ \begin{array}{l} \int_{0}^{\frac{h_{D(B)}}{2}}\sqrt{1-e^{*}{}_{D0(B0)}\left[1-\cos\left(\frac{\pi x_{0(2)}}{h_{D(B)}}\right)\right]}dx_{0(2)}=\int_{0}^{\frac{h_{D(B)}}{2}}\sqrt{1-e_{D0(B0)}\cos\left(\frac{\pi x_{0(2)}}{h_{D(B)}}\right)}dx_{0(2)}\\ \int_{0}^{\frac{h_{D(B)}}{2}}\sqrt{\alpha_{D(B)}}dx_{0(2)}=\int_{0}^{\frac{h_{D(B)}}{2}}\sqrt{1-e_{D0(B0)}\cos\left(\frac{\pi x_{0(2)}}{h_{D(B)}}\right)}dx_{0(2)} \end{array}\right.$$

Thus, the porosity and mass density coefficients can be given as listed in Table 1.

Moreover, according to the modified Halpin–Tsai model [32], *E_D_*_0(*B*0)_ can be expressed as:

(7) $$\begin{array}{ll} E_{D0(B0)}\left(x_{0(2)}\right) & =\frac{3}{8}E_{M}\left(\frac{1+\frac{E_{GPL}/E_{M}-1}{E_{GPL}/E_{M}+\xi_{lD(lB)}}\xi_{lD(lB)}V_{GPLD(GPLB)}}{1-\frac{E_{GPL}/E_{M}-1}{E_{GPL}/E_{M}+\xi_{lD(lB)}}V_{GPLD(GPLB)}}\right)\\ & +\frac{5}{8}E_{M}\left(\frac{1+\frac{E_{GPL}/E_{M}-1}{E_{GPL}/E_{M}+\xi_{wD(wB)}}\xi_{wD(wB)}V_{GPLD(GPLB)}}{1-\frac{E_{GPL}/E_{M}-1}{E_{GPL}/E_{M}+\xi_{wD(wB)}}V_{GPLD(GPLB)}}\right) \end{array}$$

in which *E_GPL_* and *E_M_* are the Young’s modulus of GPLs and the matrix, respectively. The geometry factors *ξ_lD_*_(*lB*)_ and *ξ_wD_*_(*wB*)_ of GPLs in the disk and blades are given as:

(8) $$\left\{ \begin{array}{l} \xi_{lD(lB)}=\frac{2l_{D(B)}}{t_{D(B)}}\\ \xi_{wD(wB)}=\frac{2w_{D(B)}}{t_{D(B)}} \end{array}\right.$$

where *l_D_*_(*B*)_, *w_D_*_(*B*)_, and *t_D_*_(*B*)_ are the GPL’s average length, width, and thickness in the disk and blades, respectively.

Based on the rule of mixture, *ρ_D_*_0(*B*0)_ and *μ_D_*_0(*B*0)_ are:

(9) $$\left\{ \begin{array}{l} \rho_{D0(B0)}\left(x_{0(2)}\right)=V_{GPLD(GPLB)}\rho_{GPL}+\left(1-V_{GPLD(GPLB)}\right)\rho_{M}\\ \mu_{D0(B0)}\left(x_{0(2)}\right)=V_{GPLD(GPLB)}\mu_{GPL}+\left(1-V_{GPLD(GPLB)}\right)\mu_{M} \end{array}\right.$$

in which *ρ_GPL_* and *ρ_M_* are the Young’s modulus of GPLs and the matrix, respectively; *μ_GPL_* and *μ_M_* are the Poisson’s ratio of the GPLs and the matrix, respectively.

**Figure 4:**
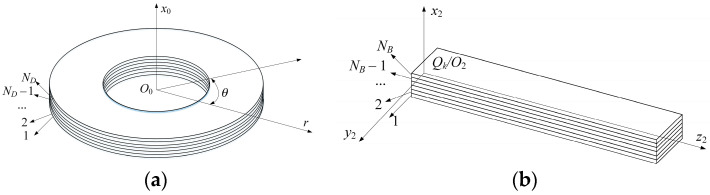
Nanocomposite structures: (**a**) for the disk, (**b**) for the *k*th blade.

Since the manufacturing of an ideal FG-GPLRC with a smooth change in material composition is impossible due to the limitation of current manufacturing technology, a multilayer structure with both GPL and porosity distributions varying from layer to layer is adopted to achieve an approximate gradient. Each layer has the same thickness and evenly distributed pores and GPLs. As shown in Figure 4, the layer numbers of the disk and blades are *N_D_* and *N_B_*, respectively.

**Figure 5:**
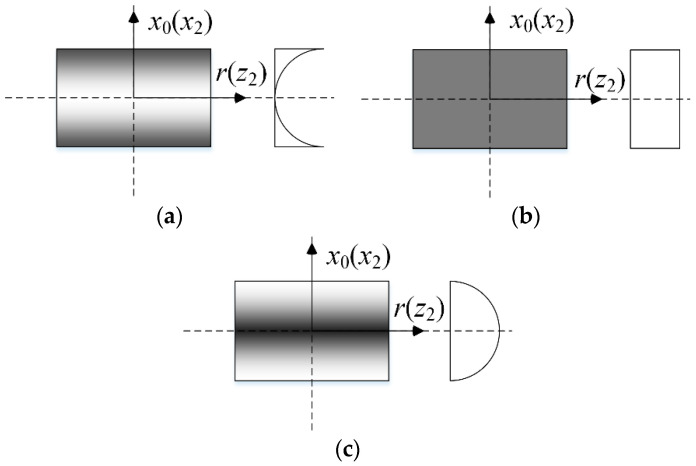
Graphene nanoplatelet (GPL) distribution patterns in the disk and blades: (**a**) Pattern X*_GD_* (X*_GB_*), (**b**) Pattern U*_GD_* (U*_GB_*), and (**c**) Pattern O*_GD_* (O*_GB_*).

Three GPL distributions shown in Figure 5 are considered in the present study. It can be found that Pattern X provides the maximum GPL volume fraction around the surfaces of the disk and blades, while Pattern O gives the minimum one. Meanwhile, Pattern U is the uniform distribution of GPLs.

For different GPL distributions, the GPL volume fraction can be written as:

(10) $$V_{GPLD(GPLB)}\left(x_{0(2)}\right)=\left\{ \begin{array}{ll} s_{q1D(q1B)}\left[1-\cos\left(\frac{\pi x_{0(2)}}{h_{D(B)}}\right)\right] & \mathrm{GPL}\;\mathrm{Pattern}\;X_{GD(GB)}\\ s_{q2D(q2B)} & \mathrm{GPL}\;\mathrm{Pattern}\;U_{GD(GB)}\\ s_{q3D(q3B)}\cos\left(\frac{\pi x_{0(2)}}{h_{D(B)}}\right) & \mathrm{GPL}\;\mathrm{Pattern}\;O_{GD(GB)} \end{array}\right.$$

where *q* = 1, 2, and 3 are corresponding to the porosity pattern X*_PD_*_(*PB*)_, U*_PD_*_(*PB*)_, and O*_PD_*_(*PB*)_; *s_q_*_1*D*(*q*1*B*)_, *s_q_*_2*D*(*q*2*B*)_, and *s_q_*_3*D*(*q*3*B*)_ are the coefficients of GPL volume fraction in the disk and blades, which can be calculated from:

(11) $$V^{T}{}_{GPLD(GPLB)}\sum_{j=1}^{N_{D(B)}}\frac{\rho_{D(B)}\left(x_{0j(2j)}\right)}{\rho_{D0(B0)}}=\left\{ \begin{array}{l} s_{q1D(q1B)}\sum_{j=1}^{N_{D(B)}}\left\{\left[1-\cos\left(\frac{\pi x_{0j(2j)}}{h_{D(B)}}\right)\right]\frac{\rho_{D(B)}\left(x_{0j(2j)}\right)}{\rho_{D0(B0)}}\right\}\\ s_{q2D(q2B)}\sum_{j=1}^{N_{D(B)}}\frac{\rho_{D(B)}\left(x_{0j(2j)}\right)}{\rho_{D0(B0)}}\\ s_{q3D(q3B)}\sum_{j=1}^{N_{D(B)}}\left[\cos\left(\frac{\pi x_{0j(2j)}}{h_{D(B)}}\right)\frac{\rho_{D(B)}\left(x_{0j(2j)}\right)}{\rho_{D0(B0)}}\right] \end{array}\right.$$

in which:

(12) $$x_{0j(2j)}=\left(\frac{1}{2}+\frac{1}{2N_{D(B)}}-\frac{j}{N_{D(B)}}\right)h_{D(B)},\;j=1,2,3,\cdots ,N_{D(B)}.$$

The total GPL volume fraction V*^T^_GPLD_*_(*GPLB*)_ is determined by:

(13) $$V^{T}{}_{GPLD(GPLB)}=\frac{W_{GPLD(GPLB)}}{W_{GPLD(GPLB)}+\rho_{GPL}\left(1-W_{GPLD(GPLB)}\right)/\rho_{M}}$$

where *W_GPLD_*_(*GPLB*)_ is the GPL weight fraction.

### 2.3. Energy Functions

The energy method is adopted to obtain the equations of motion. Thus, the kinetic energy and potential energy need to be given in the first place.

The velocity of an arbitrary point in the disk is:

(14) $$\left\{ \begin{array}{l} v_{x}={\dot{u}}_{D}\left(r,\theta \right)\\ v_{y}=\Omega r\cos\left(\Omega t+\theta \right)\\ v_{z}=-\Omega r\sin\left(\Omega t+\theta \right) \end{array}\right..$$

Thus, the kinetic energy of the disk can be obtained as:

(15) $$\begin{array}{ll} T_{D} & =\frac{1}{2}\int_{V}\rho_{D}\left({v_{x}}^{2}+{v_{y}}^{2}+{v_{z}}^{2}\right)dV\\ & =\frac{\pi }{4}\left(b^{4}-a^{4}\right)\Omega^{2}\int_{-\frac{h_{D}}{2}}^{\frac{h_{D}}{2}}\rho_{D}dx_{0}+\frac{1}{2}\int_{0}^{2\pi }\int_{a}^{b}\int_{-\frac{h_{D}}{2}}^{\frac{h_{D}}{2}}\rho_{D}{{\dot{u}}_{D}}^{2}rdx_{0}drd\theta \end{array}.$$

Considering the gyroscopic effect, on the basis of Kirchhoff’s plate theory, the total potential energy of the disk is:

(16) $$V_{D}=\frac{1}{2}\int_{0}^{2\pi }\int_{a}^{b}\int_{-\frac{h_{D}}{2}}^{\frac{h_{D}}{2}}\frac{E_{D}x_{0}{}^{2}}{\left(1-\mu_{D}{}^{2}\right)}\left\{\begin{array}{l} {\left(\nabla^{2}u_{D}\right)}^{2}-2\left(1-\mu_{D}\right)\frac{\partial^{2}u_{D}}{\partial r^{2}}\left(\frac{1}{r}\frac{\partial u_{D}}{\partial r}+\frac{1}{r^{2}}\frac{\partial^{2}u_{D}}{\partial \theta^{2}}\right)\\ +2\left(1-\mu_{D}\right){\left[\frac{\partial }{\partial r}\left(\frac{1}{r}\frac{\partial u_{D}}{\partial \theta }\right)\right]}^{2}+N_{r}^{\prime }{\left(\frac{\partial u_{D}}{\partial r}\right)}^{2}+N_{\theta }^{\prime }{\left(\frac{1}{r}\frac{\partial u_{D}}{\partial \theta }\right)}^{2} \end{array}\right\}rdx_{0}drd\theta$$

in which:

(17) $$\left\{ \begin{array}{l} \nabla^{4}u_{D}=\nabla^{2}\left(\nabla^{2}u_{D}\right),\nabla^{2}=\frac{\partial^{2}}{\partial r^{2}}+\frac{\partial }{r\partial r}+\frac{\partial^{2}}{r^{2}\partial \theta^{2}}\\ N_{r}^{\prime }=\frac{\rho_{D}\Omega^{2}}{8}\left[-\left(3+\mu_{D}\right)r^{2}+C_{1}+C_{2}\frac{1}{r^{2}}\right]\\ N_{\theta }^{\prime }=\frac{\rho_{D}\Omega^{2}}{8}\left[-\left(1+3\mu_{D}\right)r^{2}+C_{1}-C_{2}\frac{1}{r^{2}}\right] \end{array}\right.$$

where:

(18) $$\left\{ \begin{array}{l} C_{1}=\frac{\left(1+\mu_{D}\right)\left(3+\mu_{D}\right)b^{4}+\left(1-\mu_{D}{}^{2}\right)a^{4}}{\left(1+\mu_{D}\right)b^{2}+\left(1-\mu_{D}\right)a^{2}}\\ C_{2}=b^{2}a^{2}\frac{\left(1-\mu_{D}\right)\left(3+\mu_{D}\right)b^{2}-\left(1-\mu_{D}{}^{2}\right)a^{2}}{\left(1+\mu_{D}\right)b^{2}+\left(1-\mu_{D}\right)a^{2}} \end{array}\right..$$

Similarly, the velocity of an arbitrary point in the *k*th blade (*k* = 1, 2) is:

(19) $$\begin{array}{l} \left\{ \begin{array}{ll} v_{x} & ={\dot{u}}_{D}\left(Q_{k}\right)-\frac{\partial {\dot{u}}_{D}\left(Q_{k}\right)}{\partial r}\left(x_{2}+u_{Bk}\right)\sin\left({u^{\prime }}_{D}|{}_{b}\right)+\frac{\partial {\dot{u}}_{D}\left(Q_{k}\right)}{\partial r}z_{2}\cos\left(\frac{\partial u_{D}\left(Q_{k}\right)}{\partial r}\right)\\ v_{y} & =-\Omega \left(y_{2}+v_{Bk}\right)\sin\Omega t+{\dot{v}}_{Bk}\cos\phi \cos\Omega t\\ & +\Omega \left[z_{2}\cos\left(\frac{\partial u_{D}\left(Q_{k}\right)}{\partial r}\right)+\left(x_{2}+u_{Bk}\right)\sin(\frac{\partial u_{D}\left(Q_{k}\right)}{\partial r})+b\right]\cos\Omega t\\ & +\left[\begin{array}{l} {\dot{u}}_{Bk}\sin\left(\frac{\partial u_{D}\left(Q_{k}\right)}{\partial r}\right)+\frac{\partial {\dot{u}}_{D}\left(Q_{k}\right)}{\partial r}u_{Bk}\cos\left(\frac{\partial u_{D}\left(Q_{k}\right)}{\partial r}\right)\\ -z_{2}\frac{\partial {\dot{u}}_{D}\left(Q_{k}\right)}{\partial r}\sin\left(\frac{\partial u_{D}\left(Q_{k}\right)}{\partial r}\right) \end{array}\right]\cos\Omega t\\ v_{z} & =\Omega \left(y_{2}+v_{Bk}\right)\cos\Omega t+{\dot{v}}_{Bk}\sin\Omega t\\ & +\Omega \left[z_{2}\cos\left({u^{\prime }}_{D}|{}_{b}\right)+\left(x_{2}+u_{Bk}\right)\sin\left(\frac{\partial u_{D}\left(Q_{k}\right)}{\partial r}\right)+b\right]\sin\Omega t\\ & -\left[\begin{array}{l} {\dot{u}}_{Bk}\sin\left(\frac{\partial u_{D}\left(Q_{k}\right)}{\partial r}\right)+\frac{\partial {\dot{u}}_{D}\left(Q_{k}\right)}{\partial r}u_{Bk}\cos\left(\frac{\partial u_{D}\left(Q_{k}\right)}{\partial r}\right)\\ -z_{2}\frac{\partial {\dot{u}}_{D}\left(Q_{k}\right)}{\partial r}\sin\left(\frac{\partial u_{D}\left(Q_{k}\right)}{\partial r}\right) \end{array}\right]\cos\Omega t \end{array}\right. \end{array}$$

in which:

(20) $$\left\{ \begin{array}{l} {\dot{u}}_{D}\left(Q_{k}\right)={{\dot{u}}_{D}|}_{r=b,\theta =\left(k-1\right)\pi }\\ \frac{\partial {\dot{u}}_{D}\left(Q_{k}\right)}{\partial r}={\frac{\partial {\dot{u}}_{D}}{\partial r}|}_{r=b,\theta =\left(k-1\right)\pi } \end{array}\right.,\;k=1,2.$$

Therefore, the kinetic energy of the *k*th blade can be derived as:

(21) $$\begin{array}{ll} T_{Bk} & =\frac{1}{2}\int_{V}\rho_{B}(v_{x}{}^{2}+v_{y}{}^{2}+v_{z}{}^{2})dV\\ & =\frac{WL_{B}}{2}{\dot{u}}_{D}{}^{2}\left(Q_{k}\right)\int_{-\frac{h_{B}}{2}}^{\frac{h_{B}}{2}}\rho_{B}dx_{2}+\frac{WL_{B}{}^{2}}{2}{\dot{u}}_{D}\left(Q_{k}\right)\frac{\partial {\dot{u}}_{D}\left(Q_{k}\right)}{\partial r}\int_{-\frac{h_{B}}{2}}^{\frac{h_{B}}{2}}\rho_{B}dx_{2}\\ & +\frac{WL_{B}}{2}{\left[\frac{\partial {\dot{u}}_{D}\left(Q_{k}\right)}{\partial r}\right]}^{2}\int_{-\frac{h_{B}}{2}}^{\frac{h_{B}}{2}}\rho_{B}x_{2}{}^{2}dx_{2}+\frac{WL_{B}{}^{3}}{3}{\left[\frac{\partial {\dot{u}}_{D}\left(Q_{k}\right)}{\partial r}\right]}^{2}\int_{-\frac{h_{B}}{2}}^{\frac{h_{B}}{2}}\rho_{B}dx_{2}\\ & +\frac{WL_{B}}{2}\Omega^{2}{\left[\frac{\partial u_{D}\left(Q_{k}\right)}{\partial r}\right]}^{2}\int_{-\frac{h_{B}}{2}}^{\frac{h_{B}}{2}}\rho_{B}x_{2}{}^{2}dx_{2}+W{\dot{u}}_{D}\left(Q_{k}\right)\int_{-\frac{h_{B}}{2}}^{\frac{h_{B}}{2}}\rho_{B}dx_{4}\int_{0}^{L_{B}}{\dot{u}}_{Bk}dz_{2}\\ & +W\frac{\partial {\dot{u}}_{D}\left(Q_{k}\right)}{\partial r}\int_{-\frac{h_{B}}{2}}^{\frac{h_{B}}{2}}\rho_{B}dx_{2}\int_{0}^{L_{B}}z_{2}{\dot{u}}_{Bk}dz_{2}+\Omega W\frac{\partial {\dot{u}}_{D}\left(Q_{k}\right)}{\partial r}\int_{-\frac{h_{B}}{2}}^{\frac{h_{B}}{2}}\rho_{B}dx_{2}\int_{0}^{L_{B}}z_{2}v_{Bk}dz_{2}\\ & +\frac{W}{2}\int_{-\frac{h_{B}}{2}}^{\frac{h_{B}}{2}}\rho_{B}dx_{2}\int_{0}^{L_{B}}{\dot{u}}_{Bk}{}^{2}dz_{2}+\frac{W}{2}\int_{-\frac{h_{B}}{2}}^{\frac{h_{B}}{2}}\rho_{B}dx_{2}\int_{0}^{L_{B}}{\dot{v}}_{Bk}{}^{2}dz_{2}+\frac{W}{2}\Omega^{2}\int_{-\frac{h_{B}}{2}}^{\frac{h_{B}}{2}}\rho_{B}dx_{2}\int_{0}^{L_{B}}v_{Bk}{}^{2}dz_{2}\\ & +\Omega^{2}\frac{\partial u_{D}\left(Q_{k}\right)}{\partial r}W\int_{-\frac{h_{B}}{2}}^{\frac{h_{B}}{2}}\rho_{B}dx_{2}\int_{0}^{L_{B}}u_{Bk}dz_{2} \end{array}.$$

The total kinetic energy of the blades is:

(22) $$T_{B}=T_{B1}+T_{B2}.$$

According to Euler–Bernoulli beam theory, the strain of the *k*th blade is:

(23) $$\varepsilon_{z_{2}k}=-x_{2}\frac{\partial^{2}u_{Bk}}{\partial z_{2}{}^{2}}-y_{4}\frac{\partial^{2}v_{Bk}}{\partial z_{2}{}^{2}}.$$

The deformation potential energy of the *k*th blade can be written as:

(24) $$\begin{array}{ll} U_{Bk1} & =\frac{1}{2}\int_{V}E_{B}\varepsilon_{z_{2}k}{}^{2}dV\\ & =\frac{W}{2}\int_{-\frac{h_{B}}{2}}^{\frac{h_{B}}{2}}E_{B}x_{2}{}^{2}dx_{2}\int_{0}^{L_{B}}{\left(\frac{\partial^{2}u_{Bk}}{\partial z_{2}{}^{2}}\right)}^{2}dz_{2}+\frac{W^{3}}{24}\int_{-\frac{h_{B}}{2}}^{\frac{h_{B}}{2}}E_{B}dx_{2}\int_{0}^{L_{B}}{\left(\frac{\partial^{2}v_{Bk}}{\partial z_{2}{}^{2}}\right)}^{2}dz_{2} \end{array}.$$

The centrifugal force of the *k*th blade is:

(25) $$\begin{array}{ll} F_{z_{2}k} & =\int_{z_{2}}^{L_{B}}\Omega^{2}\rho_{B}\left(b+z_{2}\right)dz_{2}\\ & =\Omega^{2}\rho_{B}\left[b\left(L_{B}-z_{2}\right)+\frac{1}{2}\left(L_{B}{}^{2}-z_{2}{}^{2}\right)\right] \end{array}.$$

Thus, the centrifugal potential energy of the *k*th blade is

(26) $$\begin{array}{ll} U_{Bk2} & =\frac{1}{2}\int_{V}F_{z_{2}k}\left[{\left(\frac{\partial u_{Bk}}{\partial z_{2}}\right)}^{2}+{\left(\frac{\partial v_{Bk}}{\partial z_{2}}\right)}^{2}\right]dV\\ & =\frac{1}{2}\Omega^{2}\int_{-\frac{h_{B}}{2}}^{\frac{h_{B}}{2}}\rho_{B}dx_{2}\int_{0}^{L_{B}}\left[b\left(L_{B}-z_{2}\right)+\frac{1}{2}\left(L_{B}{}^{2}-z_{2}{}^{2}\right)\right]{\left(\frac{\partial u_{B}}{\partial z_{2}}\right)}^{2}dz_{2}\\ & +\frac{1}{2}\Omega^{2}\int_{-\frac{h_{B}}{2}}^{\frac{h_{B}}{2}}\rho_{B}dx_{2}\int_{0}^{L_{B}}\left[b\left(L_{B}-z_{2}\right)+\frac{1}{2}\left(L_{B}{}^{2}-z_{2}{}^{2}\right)\right]{\left(\frac{\partial v_{B}}{\partial z_{2}}\right)}^{2}dz_{2} \end{array}.$$

Finally, the total potential energy of the blades is:

(27) $$U_{B}=U_{B11}+U_{B12}+U_{B21}+U_{B22}.$$

### 2.4. Equations of Motion

The motion of each structure component in the double-bladed disk system is approximated by the weighted superposition of admissible functions. The displacements of the disk can be expressed as

(28) $$u_{D}\left(r,\theta ,t\right)=\cos\theta \Phi_{D}\left(r\right){\left[Q_{D}\left(t\right)\right]}^{T}$$

in which **Q***_D_*(*t*) and **Φ***_D_*(*r*) are the generalized coordinate vector and mode function vector of the disk, respectively:

(29) $$\left\{ \begin{array}{l} \Phi_{D}\left(r\right)=\left[\begin{array}{cccc} R_{1}\left(r\right) & \cdots & \begin{array}{cc} R_{j}\left(r\right) & \cdots \end{array} & R_{M}\left(r\right) \end{array}\right]\\ Q_{D}\left(t\right)=\left[\begin{array}{cccc} q_{D1}\left(t\right) & \cdots & \begin{array}{cc} q_{Dj}\left(t\right) & \cdots \end{array} & q_{DM}\left(t\right) \end{array}\right] \end{array}\right.$$

where *M* is the total mode number of the disk; the specific mode function *R_j_*(*r*) is:

(30) $$R_{j}\left(r\right)=A_{j}J_{1}\left(\beta_{j}r/b\right)+B_{j}N_{1}\left(\beta_{j}r/b\right)+C_{j}I_{1}\left(\beta_{j}r/b\right)+D_{j}K_{1}\left(\beta_{j}r/b\right)$$

in which *J*_1_ and *N*_1_ are Bessel functions of the first kind and second kind; *I*_1_ and *K*_1_ are modified Bessel functions of the first kind and second kind; *A_j_*, *B_j_*, *C_j_*, *D_j_* and *β_j_* are the coefficients determined by the boundary conditions of the disk, respectively.

In addition, the displacements of the *k*th blade are:J

(31) $$\left\{ \begin{array}{l} u_{Bk}\left(z_{3},t\right)=\Phi_{Uk}\left(z_{3}\right){\left[Q_{Uk}\left(t\right)\right]}^{T}\\ v_{Bk}\left(z_{3},t\right)=\Phi_{Vk}\left(z_{3}\right){\left[Q_{Vk}\left(t\right)\right]}^{T} \end{array}\right.$$

where (**Q***_Uk_*, **Q***_Vk_*) and (**Φ***_Uk_*, **Φ***_Vk_*) are the generalized coordinate vector and mode function vector of the *k*th blade, respectively. Their specific expressions are:

(32) $$\left\{ \begin{array}{l} Q_{Uk}\left(t\right)=\left[\begin{array}{cccc} q_{Uk1}\left(t\right) & \cdots & \begin{array}{cc} q_{Ukj}\left(t\right) & \cdots \end{array} & q_{UkN_{k}}\left(t\right) \end{array}\right]\\ Q_{Vk}\left(t\right)=\left[\begin{array}{cccc} q_{Vk1}\left(t\right) & \cdots & \begin{array}{cc} q_{Vkj}\left(t\right) & \cdots \end{array} & q_{VkP_{k}}\left(t\right) \end{array}\right] \end{array}\right.$$

(33) $$\left\{ \begin{array}{l} \Phi_{Uk}\left(z_{3}\right)=\left[\begin{array}{cccc} Y_{k1}\left(z_{3}\right) & \cdots & \begin{array}{cc} Y_{kj}\left(z_{3}\right) & \cdots \end{array} & Y_{kN_{k}}\left(z_{3}\right) \end{array}\right]\\ \Phi_{Vk}\left(z_{3}\right)=\left[\begin{array}{cccc} Y_{k1}\left(z_{3}\right) & \cdots & \begin{array}{cc} Y_{kj}\left(z_{3}\right) & \cdots \end{array} & Y_{kP_{k}}\left(z_{3}\right) \end{array}\right] \end{array}\right.$$

in which *N_k_* and *P_k_* are the total mode numbers of the *k*th blade along the *x*_3_-axis direction (*u_B_*) and *y*_3_-axis direction (*v_B_*); the specific mode function *Y_kj_*(*z*_3_) is:

(34) $$Y_{kj}\left(z_{3}\right)=\cos\alpha_{kj}z_{3}-\mathrm{ch}\alpha_{kj}z_{3}-\frac{\cos\alpha_{kj}z_{3}+\mathrm{ch}\alpha_{kj}z_{3}}{\sin\alpha_{kj}z_{3}+\mathrm{sh}\alpha_{kj}z_{3}}\left(\sin\alpha_{kj}z_{3}-\mathrm{sh}\alpha_{kj}z_{3}\right)$$

and *α_kj_* can be calculated from:

(35) $$\cos\alpha_{kj}\mathrm{ch}\alpha_{kj}+1=0.$$

Substituting the energy expressions in Section 2.3 into the Lagrange equation

(36) $$\frac{d}{dt}\left(\frac{\partial L}{\partial {\dot{q}}_{i}}\right)-\frac{\partial L}{\partial q_{i}}=0$$

gives the governing equations of coupled motion of the FG-GPLRC blade–disk system:

(37) $$M\ddot{q}\left(t\right)+G\dot{q}\left(t\right)+Kq\left(t\right)=0$$

where

(38) $$M=\left[\begin{array}{ccccc} \left(M_{D}+M_{{}_{D}}^{T}\right)/2 & M_{DU} & 0 & -M_{DU} & 0\\ M_{DU}{}^{T} & M_{U} & 0 & 0 & 0\\ 0 & 0 & M_{V} & 0 & 0\\ -M_{DU}{}^{T} & 0 & 0 & M_{U} & 0\\ 0 & 0 & 0 & 0 & M_{V} \end{array}\right]$$

(39) $$G=\left[\begin{array}{ccccc} 0 & 0 & G_{DV} & 0 & G_{DV}\\ 0 & 0 & 0 & 0 & 0\\ G_{DV}{}^{T} & 0 & 0 & 0 & 0\\ 0 & 0 & 0 & 0 & 0\\ -G_{DV}{}^{T} & 0 & 0 & 0 & 0 \end{array}\right]$$

(40) $$K=\left[\begin{array}{ccccc} K_{D1}+\left(K_{D2}+K_{D2}{}^{T}\right)/2 & K_{DU} & 0 & -K_{DU} & 0\\ K_{DU}{}^{T} & K_{U1}+K_{U2} & 0 & 0 & 0\\ 0 & 0 & K_{V1}+K_{V2}+K_{V3} & 0 & 0\\ -K_{DU}{}^{T} & 0 & 0 & K_{U1}+K_{U2} & 0\\ 0 & 0 & 0 & 0 & K_{V1}+K_{V2}+K_{V3} \end{array}\right].$$

The specific expressions of each element in the mass matrix **M**, gyroscopic matrix **G**, and stiffness matrix **K** are given in Appendix A.

Due to the gyroscopic matrix **G** caused by rotation, the coupled governing equations cannot be solved directly by the eigenvalue method. For free vibration analysis, Equation (37) needs to be converted into the state form by introducing:

(41) $$\dot{p}\left(t\right)=Bp\left(t\right)$$

in which the state vector **p**(*t*) and state matrix **B** can be determined by:

(42) $$p\left(t\right)=\left\{\begin{array}{l} q\left(t\right)\\ \dot{q}\left(t\right) \end{array}\right\},\;B=\left[\begin{array}{cc} 0 & E\\ -M^{-1}K & -M^{-1}C \end{array}\right]$$

where **E** is the unity matrix.

Setting

(43) $$p\left(t\right)=\bar{p}e^{i\omega t}$$

and substituting Equation (43) into Equation (41) yields:

(44) $$\left(B-i\omega E\right)\bar{p}=0$$

where *i* = (−1)^0.5^.

Thus, the natural frequencies *ω* of the blade–disk system can be obtained by solving the eigenvalue problem.

In addition, the backward travelling wave frequency *ω_b_* and forward travelling wave frequency *ω_f_* can be obtained as:

(45) $$\left\{ \begin{array}{l} \omega_{b}=\omega +\Omega \\ \omega_{f}=\left|\omega -\Omega \right| \end{array}\right.$$

## 3. Results and Discussion

In this section, the free vibration behavior of the spinning FG-GPLRC porous double-bladed disk system is studied comprehensively. Unless otherwise stated, the structural parameters are *a* = 0.4 m, *b* = 0.8 m, *h_D_* = 0.03m, *l_B_* = 0.4 m, *W* = 0.02 m and *h_B_* = 0.01 m; the material parameters are *E_GPL_* = 1010 GPa, *E_M_* = 130 GPa, *ρ_GPL_* = 1062.5 kg/m^3^, *ρ_M_* = 8960 kg/m^3^, *μ_GPL_* = 0.186, *μ_M_* = 0.34, *W_GPLD_* = *W_GPLB_* = 1%, *L_D_*/*t_D_* = *L_B_*/*t_B_* = 100 and *L_D_*/*B_D_* = *L_B_*/*B_B_* = 2, *e_0D_* = *e_0B_* = 0.1. Moreover, the porosity distributions pattern X*_PD_*, X*_PB_* and GPL distribution patterns X*_GD_* and X*_GB_* are considered in the following analysis.

### 3.1. Convergence and Comparison Study

Before parametric analysis, the convergence regarding the vibration mode number and GPL layer number used in the analysis is investigated firstly. As displayed in Table 2 and Table 3, convergent results for the first three frequencies can be achieved at (*M* = 5, *N*_1_ = *N*_2_ = 16, *P*_1_ = *P*_2_ = 13) and (*N_D_* = *N_B_* = 16), which will be adopted in the following calculations.

**Figure 6:**
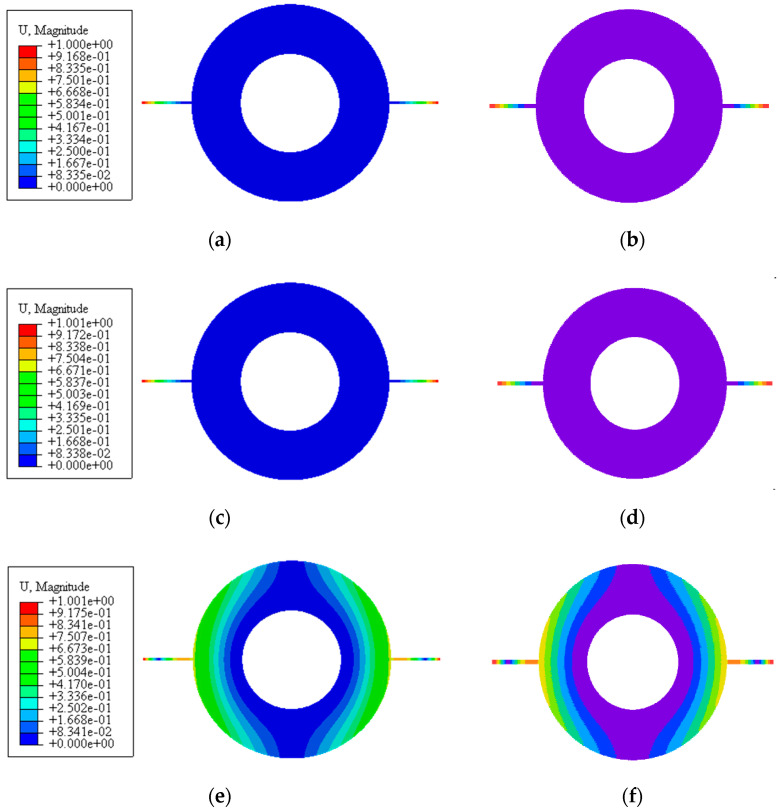
Vibration modes of the double-bladed disk system: (**a**,**c**,**e**) First three mode obtained by ABAQUS, (**b**,**d**,**f**) First three mode obtained by MATLAB.

Since there are no suitable data in the open literatures for direct comparison, the finite element method using commercial software ABAQUS is employed to further verify the present modeling and vibration analysis. Table 4 and Figure 6 show the comparison of frequencies and vibration modes between theoretical (MATLAB) and FE (ABAQUS) results with different spinning speeds, respectively. Here, GPL distributions U*_GD_* and U*_GB_* and porosity distribution patterns U*_PD_* and U*_PB_* are considered. Very good agreement is observed, which indicates that the present analysis is accurate.

### 3.2. Free Vibration Analysis

**Figure 7:**
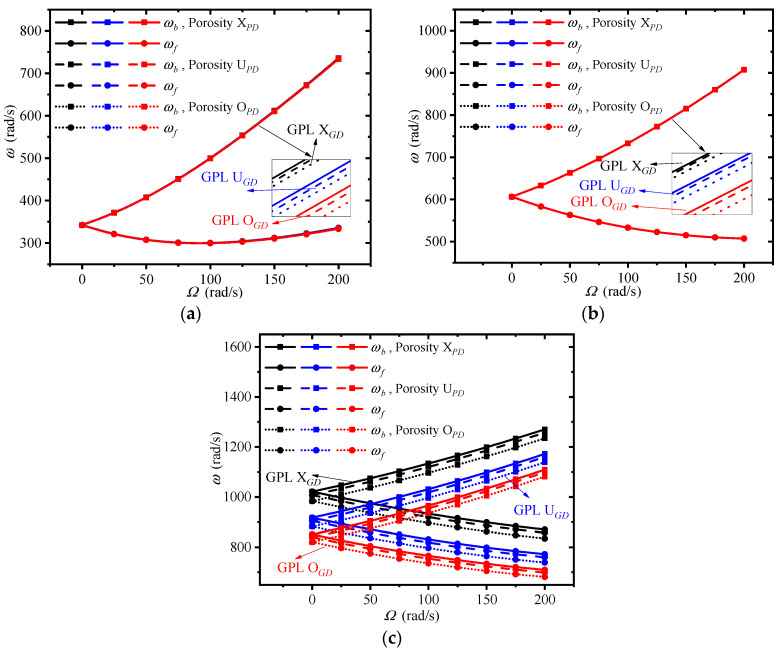
Effects of graphene nanoplatelet (GPL) and porosity distribution in the disk on the traveling wave frequencies of the double-bladed system: (**a**) First frequency, (**b**) Second frequency, (**c**) Third frequency.

In what follows, the effects of the material and structural parameters on the free vibration of the double-bladed disk are examined in detail. Figure 7 depicts the changes of first three traveling wave frequencies of the double-bladed disk system with spinning speed for different distributions of GPLs and porosity in the disk, where the distributions of GPLs and porosity in the blades remain X*_GB_* and X*_PB_*; different color lines stand for different GPL distributions; different type lines stand for different porosity distributions; two different marks in the lines stand for backward and forward traveling wave frequencies. As can be seen, the first forward traveling wave frequency decreases first and then rises, while the first backward traveling wave frequency increases monotonously. The second and third forward traveling wave frequencies are decreased continuously, while the second and third backward traveling wave frequencies grow on and on. Another observation is that the porosity distribution X*_PD_* and GPL distribution X*_GD_* provide the largest frequencies compared to other porosity and GPL distributions. It indicates that dispersing more GPLs around the surfaces of the disk is effective to improving the mechanical performance of the double-bladed disk system. Meanwhile, the non-uniform porosity distribution X*_PD_* can help to achieve the highest structural stiffness. It can be found that the GPL distribution patterns in the disk have a greater influence on the traveling wave frequencies than porosity distribution patterns. Moreover, the third traveling wave frequency is affected markedly by the GPL and porosity distribution in the disk, while the first two traveling wave frequencies are impacted little. This is because the vibration mode corresponding to the third frequency contains obvious disk vibration, as shown in Figure 6.

**Figure 8:**
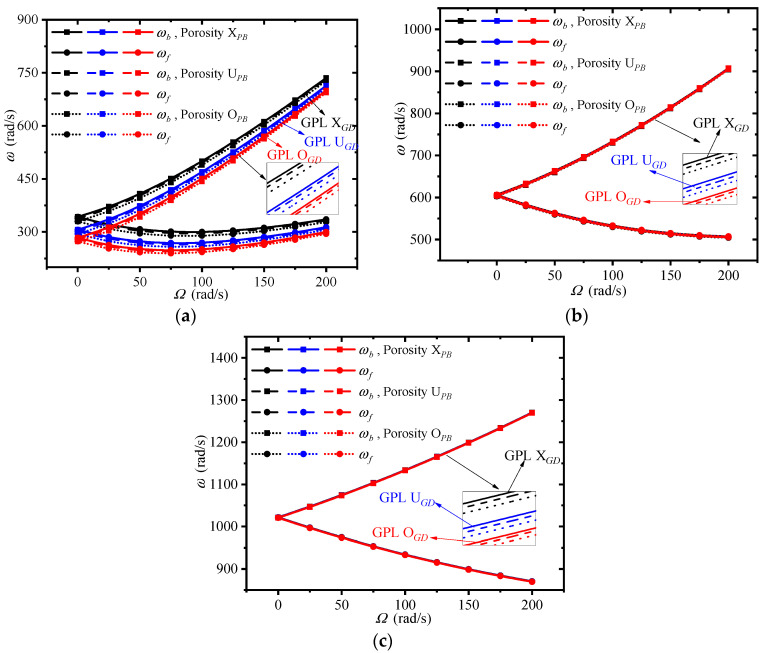
Effects of GPL and porosity distribution in the disk on the traveling wave frequencies of the double-bladed system: (**a**) First frequency, (**b**) Second frequency, (**c**) Third frequency.

Figure 8 plots the changes of first three traveling wave frequencies of the double-bladed disk system with spinning speed for different distributions of GPLs and porosity in the blades, where the distributions of GPLs and porosity in the disk remain X*_GD_* and X*_PD_*. It can be seen that the rotor system with porosity distribution X*_PB_* and GPL distribution X*_GB_* has largest traveling wave frequencies.

This implies that adding more GPLs around the surfaces of the blades has an obvious advantage to enhance the rotor stiffness. It can be told that the non-uniform pattern X*_PB_* is the best candidate among the presented porosity distributions. The traveling wave frequencies are more sensitive to the GPL distribution patterns in the blades than porosity distributions. In addition, the GPL and porosity distribution in the blades mostly affect the first traveling wave frequency, which is because the vibration mode corresponding to the first frequency is mainly reflected by the blades’ displacements along their thickness direction.

Since the variations of forward and backward traveling frequencies exhibit quite a similar trend, only the backward traveling frequencies under two typical spinning speeds—0 rad/s and 100 rad/s—are listed in Table 5, Table 6, Table 7 and Table 8.

Table 5 shows the first three traveling wave frequencies of the double-bladed disk system for different GPL weight fractions, where (*p* = 0, *q* = *f*), (*p* = *f*, *q* = 0), and (*p* = *q* = *f*) stand for only blades are reinforced, only disk is reinforced, and both blades and disk are equally reinforced by GPLs. A considerable rise in the traveling wave frequencies is observed as the GPL weight fraction increases, which indicates that dispersing more GPLs at a low total content can achieve better mechanical performance of the double-bladed disk system. In addition, the first two frequencies are mainly influenced by the GPL weight fraction in the blades, while the third frequency is mostly affected by that in the disk.

Table 6 lists the first three traveling wave frequencies of the double-bladed disk system for different GPL length-to-thickness ratios. A higher GPL length-to-thickness ratio leads to increased traveling wave frequencies. For the same content of GPLs, larger values of GPL length-to-thickness ratio mean thinner GPLs. Thus, it can be noted that adopting thinner GPLs is effective in improving the vibration behavior of double-bladed disk system. Similar to the GPL weight fraction, the first two frequencies are more sensitive to the GPL length-to-thickness ratio in the blades.

Table 7 gives the first three traveling wave frequencies of the double-bladed disk system for different GPL length-to-width ratios, where GPL length remains constant. Actually, lower GPL length-to-width ratios represent GPLs with larger surface areas. It is obvious that the traveling wave frequencies decrease with the increase of the GPL length-to-width ratio. This is because larger surface contact areas between the matrix and GPLs lead to better load transfer capacity. In addition, the first two frequencies and third frequency are primarily impacted by the GPL length-to-width ratio in the blades and disk, respectively.

Table 8 shows the first three traveling wave frequencies of the double-bladed disk system for different porosity coefficients. It is seen that the traveling wave frequencies are reduced in general with an increase in the porosity coefficient. A greater porosity coefficient means more and larger pores in the matrix, which weaken the stiffness of the double-bladed disk system, and the first two frequencies and third frequency are mainly determined by the porosity coefficient in the blades and disk, respectively. However, an interesting phenomenon occurs in third frequency (*p* = 0, *q* = *f*): the frequency has a slight rise when the porosity coefficient in the blades increases solely.

**Figure 9:**
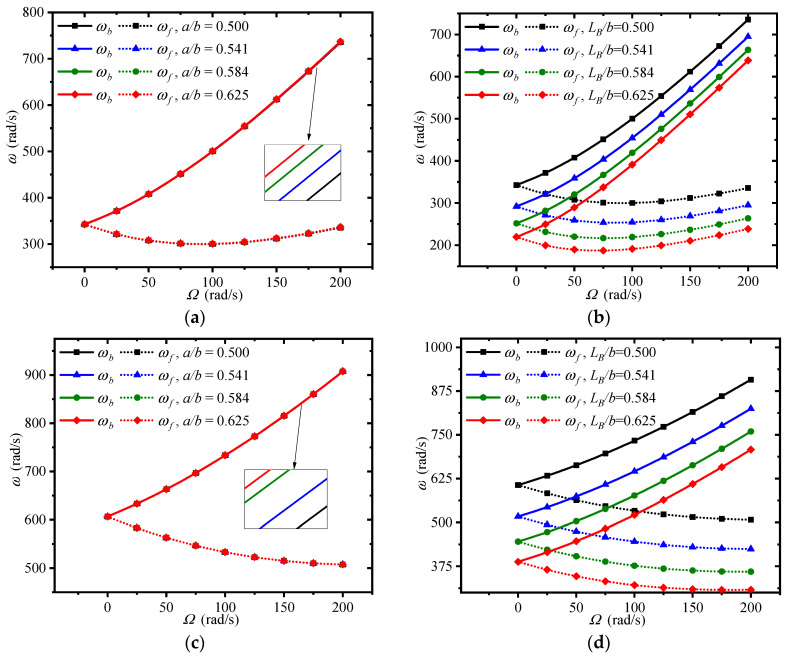

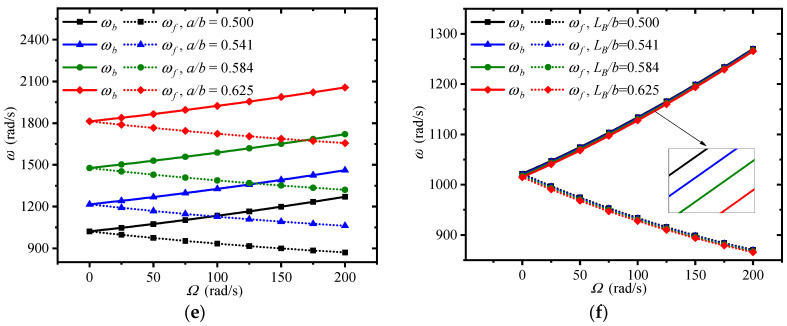
Effects of disk inner radius and blade length on the traveling wave frequencies of the double-bladed system: (**a**,**b**) First frequency, (**c**,**d**) Second frequency, (**e**,**f**) Third frequency.

Figure 9 plots the changes of first three traveling wave frequencies of the double-bladed disk system with spinning speeds for different disk inner radiuses and blade lengths, where the disk outer radius keeps constant. Increasing the disk inner radius and decreasing the blade length results in the rise of the traveling wave frequencies. It implies that a larger disk inner radius and shorter blade length in comparison with the disk outer radius should be designed to achieve better structural stiffness. Moreover, it can be found that the disk inner radius majorly influences the third frequency, while the blade length chiefly affects the first two frequencies.

## 4. Conclusions

In this paper, the coupled free vibration behavior of a spinning FG-GPLRC porous double-bladed disk system has been investigated for the first time. Based on Euler–Bernoulli beam theory and Kirchhoff plate theory, the equations of motion are derived by adopting the Lagrange equation method, which are solved by the substructure mode synthesis method and the assumed modes method. It is found from numerical results that using non-uniform porosity patterns X*_PD_* and X*_PB_* and dispersing more GPLs near the surfaces of the disk and blades are the most effective way to improve the structural stiffness and mechanical performance of the double-bladed disk system. The first traveling wave frequency is primarily influenced by the GPL and porosity distribution in the blades, while the third traveling wave frequency is remarkedly affected by those in the disk. A better reinforcement effect can be achieved when thinner GPLs with larger surface areas are used. In general, the traveling wave frequencies become lower at a higher porosity. However, the third-order frequency increases slightly when only the porosity in the blades increases. In addition, the first two frequencies are considerably influenced by the weight fraction, length-to-thickness ratio, and length-to-width ratio of GPLs, porosity coefficient, and the length of the blades, while the third-order frequency is mainly affected by those factors and the inner radius of the disk.

## Figures and Tables

**Table 1:** Variation of porosity coefficients.

*e*_0_	*e*_1_	*e*_2_
0.1	0.1738	0.9361
0.2	0.3442	0.8716
0.3	0.5103	0.8064
0.4	0.6708	0.7404

**Table 2:** Frequencies *ω* (rad/s) of the functionally graded graphene nanoplatelets reinforced composite (FG-GPLRC) porous double-bladed disk system with different mode numbers (*Ω* = 0 rad/s).

Frequency	*M* = 4 *N*_1_ = *N*_2_ = 14 *P*_1_ = *P*_2_ = 11	*M* = 5 *N*_1_ = *N*_2_ = 16 *P*_1_ = *P*_2_ = 13	*M* = 5 *N*_1_ = *N*_2_ = 16 *P*_1_ = *P*_2_ = 15	*M* = 5 *N*_1_ = *N*_2_ = 18 *P*_1_ = *P*_2_ = 13	*M* = 6 *N*_1_ = *N*_2_ = 16 *P*_1_ = *P*_2_ = 13
First	342.50824	342.41852	342.41852	342.41842	342.41559
Second	606.54032	606.39077	606.39076	606.39077	606.39077
Third	1021.84498	1021.61920	1021.61920	1021.61920	1021.61826
Fourth	2149.98662	2149.78629	2149.78629	2149.78492	2149.78629
Fifth	3801.04613	3800.18608	3800.18590	3800.18608	3800.18608

**Table 3:** Frequencies *ω* (rad/s) of the FG-GPLRC porous double-bladed disk system with different layer numbers (*Ω* = 0 rad/s).

Frequency	*N_D_* = *N_B_* = 4	*N_D_* = *N_B_* = 8	*N_D_* = *N_B_* = 16	*N_D_* = *N_B_* = 100
First	333.419	340.665	342.418	342.982
Second	605.834	606.284	606.390	606.424
Third	995.799	1016.59	1021.61	1023.23
Fourth	2093.27	2138.77	2149.78	2153.33
Fifth	3796.69	3799.51	3800.18	3800.40

**Table 4:** Frequencies *ω* (rad/s) of the FG-GPLRC blade–disk system: comparison between theoretical and finite element results.

Frequency	*Ω* (rad/s)	Present	Finite Element	Error
First	0	301.7845	296.1836	1.84%
	100	604.6508	594.0126	1.74%
	200	904.2086	900.3008	0.38%
Second	0	365.8797	167.3024	1.51%
	100	432.1137	425.2251	1.62%
	200	918.1062	914.1168	0.39%
Third	0	509.8145	503.8130	1.14%
	100	705.8883	695.0076	1.51%
	200	959.1757	958.6897	0.44%

**Table 5:** Effect of GPL weight fraction on the backward traveling frequencies (rad/s) of the double-bladed system at different spinning speeds (rad/s).

Frequency	*Ω*	*W_GPLD_* = *p* *W_GPLB_* = *q*	*f* = 0%	*f* = 0.33%	*f* = 0.67%	*f* = 1%
First	0	*p* = 0, *q* = *f*	240.683	278.0561	311.9853	341.7586
		*p* = *f*, *q* = 0	240.683	240.7882	240.8503	240.8894
		*p* = *q* = *f*	240.683	278.2255	312.3795	342.4185
	100	*p* = 0, *q* = *f*	417.1995	446.2748	473.9031	498.9130
		*p* = *f*, *q* = 0	417.1995	417.4847	417.6507	417.7542
		*p* = *q* = *f*	417.1995	446.6563	474.6823	500.1056
Second	0	*p* = 0, *q* = *f*	475.4040	522.0961	566.3625	606.3908
		*p* = *f*, *q* = 0	475.4040	475.4040	475.4040	475.4040
		*p* = *q* = *f*	475.4040	522.0961	566.3625	606.3908
	100	*p* = 0, *q* = *f*	609.1708	653.0075	694.9589	733.1512
		*p* = *f*, *q* = 0	609.1708	609.1910	609.2009	609.2066
		*p* = *q* = *f*	609.1708	653.0347	695.0128	733.2381
Third	0	*p* = 0, *q* = *f*	724.8524	725.4668	726.0604	726.6336
		*p* = *f*, *q* = 0	724.8524	834.5594	933.3375	1019.349
		*p* = *q* = *f*	724.8524	835.2214	934.7485	1021.619
	100	*p* = 0, *q* = *f*	842.1417	842.7876	843.4427	844.0990
		*p* = *f*, *q* = 0	842.1417	949.6112	1046.8747	1131.864
		*p* = *q* = *f*	842.1417	950.2492	1048.1906	1133.928

**Table 6:** Effect of GPL length-to-thickness ratio on the backward traveling frequencies (rad/s) of the double-bladed system at different spinning speeds (rad/s).

Frequency	*Ω*	*l_D_/t_D_* = *p* *l_B_/t_B_* = *q*	*f* = 10	*f* = 40	*f* = 70	*f* = 100
First	0	*p* = 10, *q* = *f*	320.7607	337.5028	340.8724	342.3215
		*p* = *f*, *q* = 10	320.7607	320.8224	320.8336	320.8383
		*p* = *q* = *f*	320.7607	337.5763	340.9623	342.4185
	100	*p* = 10, *q* = *f*	481.7063	495.8319	498.6986	499.9337
		*p* = *f*, *q* = 10	481.7063	481.8224	481.8435	481.8523
		*p* = *q* = *f*	481.7063	495.9637	498.8583	500.1056
Second	0	*p* = 10, *q* = *f*	578.3558	600.044	604.4773	606.3908
		*p* = *f*, *q* = 10	578.3558	578.3558	578.3558	578.3558
		*p* = *q* = *f*	578.3558	600.044	604.4773	606.3908
	100	*p* = 10, *q* = *f*	706.4411	727.1565	731.3988	733.2307
		*p* = *f*, *q* = 10	706.4411	706.4463	706.4472	706.4476
		*p* = *q* = *f*	706.4411	727.1622	731.4057	733.2381
Third	0	*p* = 10, *q* = *f*	957.0838	957.3238	957.3725	957.3935
		*p* = *f*, *q* = 10	957.0838	1006.949	1016.991	1021.310
		*p* = *q* = *f*	957.0838	1007.189	1017.28	1021.619
	100	*p* = 10, *q* = *f*	1070.217	1070.468	1070.519	1070.541
		*p* = *f*, *q* = 10	1070.217	1119.428	1129.346	1133.613
		*p* = *q* = *f*	1070.217	1119.673	1129.640	1133.928

**Table 7:** Effect of GPL length-to-width ratio on the backward traveling frequencies (rad/s) of the double-bladed system at different spinning speeds (rad/s).

Frequency	*Ω*	*l_D_/w_D_* = *p* *l_B_/w_B_* = *q*	*f* = 2	*f* = 4	*f* = 6	*f* = 8
First	0	*p* = 1, *q* = *f*	342.4185	339.9449	337.7323	335.7413
		*p* = *f*, *q* = 1	342.4185	342.4085	342.3992	342.3908
		*p* = *q* = *f*	342.4185	339.9351	337.7139	335.7154
	100	*p* = 1, *q* = *f*	500.1056	497.9969	496.1141	494.4228
		*p* = *f*, *q* = 1	500.1056	500.0878	500.0715	500.0565
		*p* = *q* = *f*	500.1056	497.9794	496.0812	494.3762
Second	0	*p* = 1, *q* = *f*	606.3908	603.1327	600.2314	597.6311
		*p* = *f*, *q* = 1	606.3908	606.3908	606.3908	606.3908
		*p* = *q* = *f*	606.3908	603.1327	600.2314	597.6311
	100	*p* = 1, *q* = *f*	733.2381	730.1191	727.3429	724.8556
		*p* = *f*, *q* = 1	733.2381	733.2374	733.2367	733.2361
		*p* = *q* = *f*	733.2381	730.1184	727.3415	724.8537
Third	0	*p* = 1, *q* = *f*	1021.619	1021.584	1021.552	1021.524
		*p* = *f*, *q* = 1	1021.619	1014.254	1007.666	1001.74
		*p* = *q* = *f*	1021.619	1014.219	1007.6	1001.644
	100	*p* = 1, *q* = *f*	1133.928	1133.891	1133.859	1133.830
		*p* = *f*, *q* = 1	1133.928	1126.653	1120.147	1114.295
		*p* = *q* = *f*	1133.928	1126.616	1120.078	1114.197

**Table 8:** Effect of porosity coefficient on the backward traveling frequencies (rad/s) of the double-bladed system at different spinning speeds (rad/s).

Frequency	*Ω*	*l_D_/w_D_* = *p* *l_B_/w_B_* = *q*	*f* = 0	*f* = 0.1	*f* = 0.2	*f* = 0.3
First	0	*p* = 0, *q* = *f*	343.4377	342.4425	341.5784	340.8869
		*p* = *f*, *q* = 0	343.4377	343.4126	343.3854	343.3555
		*p* = *q* = *f*	343.4377	342.4185	341.5304	340.8149
	100	*p* = 0, *q* = *f*	500.9792	500.1474	499.4285	498.8582
		*p* = *f*, *q* = 0	500.9792	500.9357	500.8884	500.8366
		*p* = *q* = *f*	500.9792	500.1056	499.3448	498.7324
Second	0	*p* = 0, *q* = *f*	615.0141	606.3908	597.4517	588.1804
		*p* = *f*, *q* = 0	615.0141	615.0141	615.0141	615.0141
		*p* = *q* = *f*	615.0141	606.3908	597.4517	588.1804
	100	*p* = 0, *q* = *f*	741.5002	733.2395	724.6866	715.8272
		*p* = *f*, *q* = 0	741.5002	741.4987	741.4970	741.4952
		*p* = *q* = *f*	741.5002	733.2381	724.6838	715.8232
Third	0	*p* = 0, *q* = *f*	1024.607	1024.751	1024.905	1025.070
		*p* = *f*, *q* = 0	1024.607	1021.471	1018.718	1016.468
		*p* = *q* = *f*	1024.607	1021.619	1019.033	1016.975
	100	*p* = 0, *q* = *f*	1136.880	1136.997	1137.122	1137.257
		*p* = *f*, *q* = 0	1136.880	1133.809	1131.118	1128.928
		*p* = *q* = *f*	1136.880	1133.928	1131.372	1129.338

## Appendix A

The specific expressions of each element in the mass matrix **M**, gyroscopic matrix **G**, and stiffness matrix **K**.

$$\begin{array}{ll} M_{D} & =2\pi \int_{-\frac{h_{D}}{2}}^{\frac{h_{D}}{2}}\rho_{D}dx_{0}\int_{a}^{b}r\Phi_{D}^{T}\Phi_{D}dr+2WL_{B}\Phi_{D}^{T}|{}_{b}\Phi_{D}|{}_{b}\int_{-\frac{h_{B}}{2}}^{\frac{h_{B}}{2}}\rho_{B}dx_{2}+2WL_{B}{}^{2}{\Phi^{\prime }}_{D}^{T}|{}_{b}\Phi_{D}|{}_{b}\int_{-\frac{h_{B}}{2}}^{\frac{h_{B}}{2}}\rho_{B}dx_{2}\\ & +2\left(WL_{B}\int_{-\frac{h_{B}}{2}}^{\frac{h_{B}}{2}}\rho_{B}x_{2}{}^{2}dx_{2}+\frac{2WL_{B}{}^{2}}{3}\int_{-\frac{h_{B}}{2}}^{\frac{h_{B}}{2}}\rho_{B}dx_{2}\right){\Phi^{\prime }}_{D}^{T}|{}_{b}{\Phi^{\prime }}_{D}|{}_{b} \end{array}$$

$$M_{DU}=W\Phi_{D}^{T}|{}_{b}\int_{-\frac{h_{B}}{2}}^{\frac{h_{B}}{2}}\rho_{B}dx_{2}\int_{0}^{L_{B}}\Phi_{U}dz_{2}+W{\Phi^{\prime }}_{D}^{T}|{}_{b}\int_{-\frac{h_{B}}{2}}^{\frac{h_{B}}{2}}\rho_{B}dx_{2}\int_{0}^{L_{B}}z_{2}\Phi_{U}dz_{2}$$

$$M_{U}=W\int_{-\frac{h_{B}}{2}}^{\frac{h_{B}}{2}}\rho_{B}dx_{2}\int_{0}^{L_{B}}\Phi_{U}^{T}\Phi_{U}dz_{2},\;M_{V}=W\int_{-\frac{h_{B}}{2}}^{\frac{h_{B}}{2}}\rho_{B}dx_{2}\int_{0}^{L_{B}}\Phi_{V}^{T}\Phi_{V}dz_{2},$$

$$G_{DV}=\cos\phi \Omega W{\Phi^{\prime }}_{D}^{T}|{}_{b}\;\int_{-\frac{h_{B}}{2}}^{\frac{h_{B}}{2}}\rho_{B}dx_{2}\int_{0}^{l_{B}}\;z_{2}\Phi_{V}dz_{2},$$

$$\begin{array}{ll} K_{D2} & =\pi \int_{a}^{b}\int_{-\frac{h_{D}}{2}}^{\frac{h_{D}}{2}}\{\frac{E_{D}x_{0}{}^{2}}{1-\mu_{D}{}^{2}}\;r\;\Phi_{D}^{{}^{\prime \prime }T}\Phi_{D}^{\prime \prime }+2\frac{E_{D}x_{0}{}^{2}}{1-\mu_{D}{}^{2}}\mu_{D}\;\Phi_{D}^{{}^{\prime \prime }T}\Phi_{D}^{\prime }+\frac{E_{D}x_{0}{}^{2}}{1-\mu_{D}{}^{2}}\frac{\left(3-2\mu_{D}\right)}{r}\Phi_{D}^{{}^{\prime }T}\Phi_{D}^{\prime }\\ & +\frac{\rho_{D}\Omega^{2}}{8}[-\left(3+\mu_{D}\right)r^{3}+C_{1}r+C_{2}\frac{1}{r}]\Phi_{D}^{{}^{\prime }T}\Phi_{D}^{\prime }+\frac{E_{D}x^{2}}{1-\mu_{D}{}^{2}}\frac{\left(3-2\mu_{D}\right)}{r^{3}}\Phi_{D}^{T}\Phi_{D}\\ & +\frac{\rho_{D}\Omega^{2}}{8}[-\left(1+3\mu_{D}\right)r+C_{1}\frac{1}{r}-C_{2}\frac{1}{r^{3}}]\Phi_{D}^{T}\Phi_{D}\\ & -2\frac{E_{D}x_{0}{}^{2}}{1-\mu_{D}{}^{2}}\frac{\;\mu_{D}}{r}\Phi_{D}^{{}^{\prime \prime }T}\Phi_{D}-2\frac{E_{D}x_{0}{}^{2}}{1-\mu_{D}{}^{2}}\frac{\left(3-2\mu_{D}\right)}{r^{2}}\Phi_{D}^{{}^{\prime }T}\;\Phi_{D}\}dx_{0}dr \end{array}$$

$$K_{D1}=-2\Omega^{2}{\Phi^{\prime }}_{D}^{T}|{}_{b}{\Phi^{\prime }}_{D}|{}_{b}WL_{B}\int_{-\frac{h_{B}}{2}}^{\frac{h_{B}}{2}}\rho_{B}x_{2}{}^{2}dx_{2}\;K_{U}=W\int_{-\frac{h_{B}}{2}}^{\frac{h_{B}}{2}}E_{B}x_{2}{}^{2}dx_{2}\int_{0}^{L_{B}}\Phi_{U}^{{}^{\prime \prime }T}\;\Phi_{U}^{\prime \prime }dz_{2},$$

$$K_{U2}=\Omega^{2}W\int_{-\frac{h_{B}}{2}}^{\frac{h_{B}}{2}}\rho_{B}dx_{2}\int_{0}^{L_{B}}\left[b\left(L_{B}-z_{2}\right)+\frac{1}{2}\left(L_{B}{}^{2}-z_{2}{}^{2}\right)\right]\Phi_{U}^{{}^{\prime }T}\;\Phi_{U}^{\prime }dz_{2}$$

$$K_{V2}=\Omega^{2}W\int_{-\frac{h_{B}}{2}}^{\frac{h_{B}}{2}}\rho_{B}dx_{2}\int_{0}^{L_{B}}\left[b\left(L_{B}-z_{2}\right)+\frac{1}{2}\left(L_{B}{}^{2}-z_{2}{}^{2}\right)\right]\Phi_{V}^{{}^{\prime }T}\;\Phi_{V}^{\prime }dz_{2}$$

$$K_{V}=\frac{W^{3}}{12}\int_{-\frac{h_{B}}{2}}^{\frac{h_{B}}{2}}E_{B}dx_{2}\int_{0}^{L_{B}}\Phi_{V}^{{}^{\prime \prime }T}\Phi_{V}^{\prime \prime }dz_{2},\;K_{V3}=-\Omega^{2}M_{V},$$

$$K_{DU}=-\Omega^{2}W{\Phi^{\prime }}_{D}^{T}|{}_{b}\;\int_{-\frac{h_{B}}{2}}^{\frac{h_{B}}{2}}\rho_{B}dx_{2}\int_{0}^{L_{B}}\;z_{2}\Phi_{U}dz_{2}$$
